# Suanzaoren decoction exerts its antidepressant effect via the CaMK signaling pathway

**DOI:** 10.1515/tnsci-2022-0341

**Published:** 2024-05-08

**Authors:** Xiaofang Zhang, Jiyuan Guo, Ce Zhang, Wenhua Wang, Shuailin Du, Xusheng Tian

**Affiliations:** Graduate School of Heilongjiang University of Chinese Medicine, Harbin, China; Heilongjiang University of Chinese Medicine, Harbin, China

**Keywords:** depression, Suanzaoren decoction, hippocampus, CaMK signaling pathway

## Abstract

Calmodulin-dependent protein kinases (CaMKs) are widely regarded as “memory molecules” due to their role in controlling numerous neuronal functions in the brain, and the CaMK signaling pathway plays a crucial role in controlling synaptic plasticity. Suanzaoren decoction (SZRD) can improve depression-like behavior and thus has potential benefits in the clinical treatment of depression; however, its mechanism of action is not fully understood. In this study, we found that key proteins in the CaMK signaling pathway were regulated by the decoction used to treat depression. The purpose of this research was to ascertain if the SZRD’s therapeutic efficacy in the treatment of depression is associated with the modulation of key proteins in the CaMK signaling pathway. A rat model of depression was created by exposing the animals to chronic, unexpected, mild stress. Model rats were given intragastric administration of SZRD or fluoxetine every morning once a day. Protein and mRNA relative expression levels of CaM, CaMK I, and CaMK IV in the hippocampus were measured by Western blot, quantitative polymerase chain reaction, and immunohistochemistry in the hippocampus. Our findings demonstrated that SZRD significantly improved the mood of depressed rats. This indicates that SZRD, by modulating the CaMK signaling system, may alleviate depressive symptoms and lessen work and life-related pressures.

## 1 Introduction

Depression is a comprehensive psychological disease characterized by symptoms of anhedonia and poor emotional regulation, which is often moderated by stress [1]. It has a high rate of occurrence and recurrence and can cause significant impairment, although there can be intermittent periods of complete remission, and some patients have residual symptoms even after treatment. According to the WHO’s updated International Classification of Diseases (ICD-11) published in 2018, the depressive symptoms of patients are mainly low mood or lack of interest and pleasure lasting for 2 weeks or more, accompanied by anxiety symptoms for 6 months. These symptoms can lead to varying degrees of cognitive and behavioral changes, and they are often accompanied by sleep disorders, appetite disturbances, suicidal behavior, etc. [2,3]. The WHO ranked depression as the world’s third leading cause of disability in 2008, and depression became the second most common disease in the world in 2020, and it is predicted that by 2030, the incidence of the disease will be ranked first [4]. Clinical symptoms and scales remain the primary basis for depression diagnosis, regardless of treatment modality, and objective indicators and experimental diagnoses remain unavailable [5,6]. Existing antidepressants have serious side effects, slow clinical onset, easy relapse after drug withdrawal, insufficient treatment response, a lag in treatment between administration and symptom relief, and related safety issues, which limit the scope of clinical use of traditional antidepressants [7]. In addition, inadequate awareness of or prejudice against depression prevents a significant proportion of patients from receiving professional treatment, with data showing that 76–85% of the patients do not receive treatment, and in some countries, less than 10% receive treatment [8]. According to epidemiological investigations, about 50% of the patients see improvement in their symptoms or even return to their pre-disease state after treatment with antidepressants or evidence-based psychotherapy [9], while about 30% of the patients still show a risk of recurrence and more than 15% of the patients experience no benefit from antidepressant drugs [10].

Ca^2+^/calmodulin-dependent protein kinase (CaMK) is a Ser/Thr kinase that activates CaM by extracellular stimuli (hormones, neurotransmitters, etc.) that cause an increase in intracellular Ca^2+^ concentrations [11]. CaMK, like other protein kinases, influences functions, including enzymatic efficiency, receptor activity, cytoskeletal organization, and transcriptional regulation by phosphorylating specific residues in particular cellular proteins. Physiologically limited enzymes and multifunctional CaMKs with diverse substrate specificity can be distinguished by the way in which they target their respective substrates. On the other hand, multifunctional CaMKs, including CaMK I, CaMK II, and CaMK IV, can act on multiple cellular proteins to transduce Ca^2+^ signaling to cellular physiology [12,13]. Regardless of CaMKs’ substrate specificity, their molecular structure is similar.

**Figure 1:**
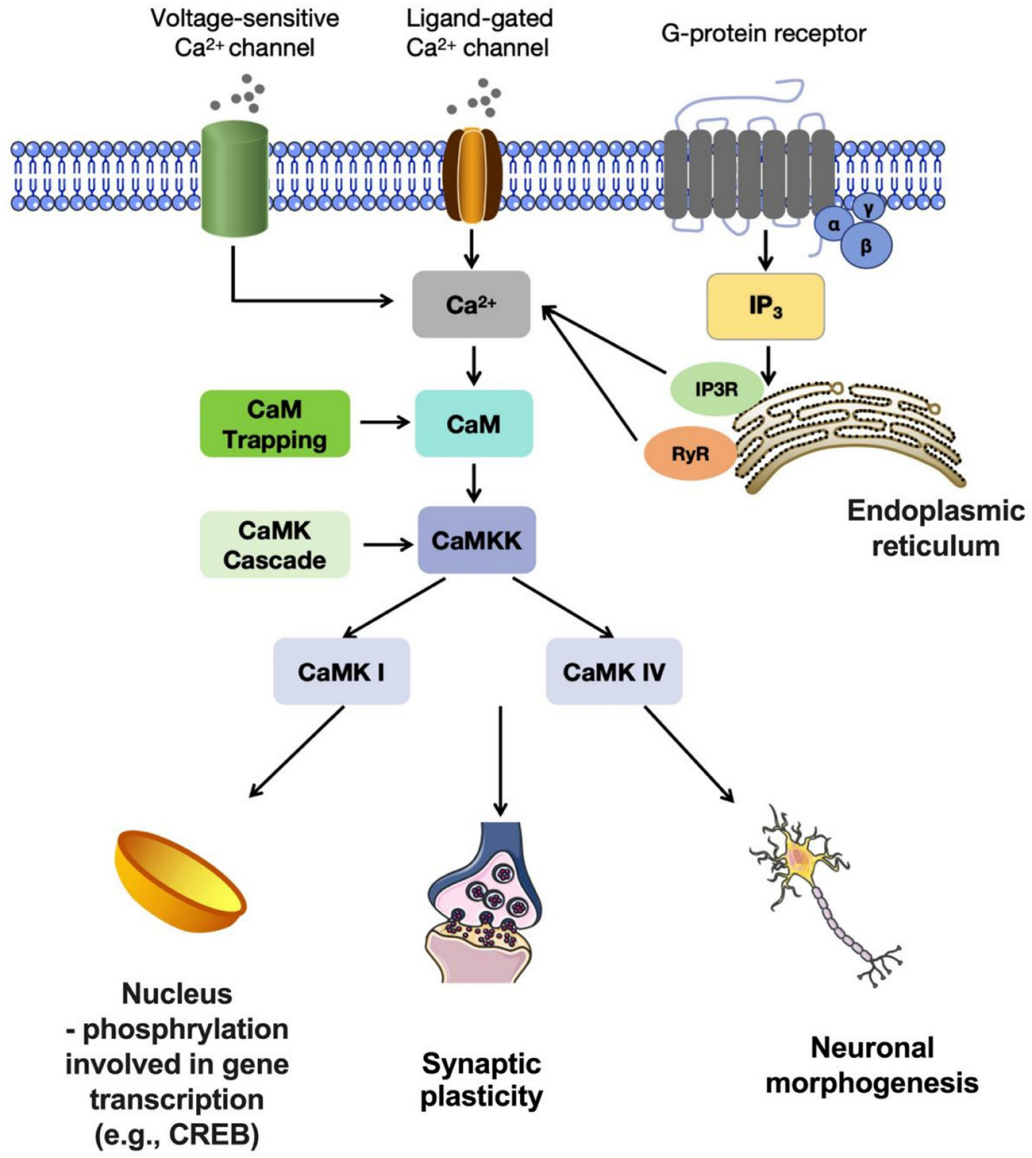
The CaM, CaMK I, and CaMK IV activation cascades and function in the central nervous system. The concentration of Ca^2+^ in the cytoplasm increases when voltage-sensitive Ca^2+^ channels, ligand-gated ionotropic channels, or G-protein-coupled receptors are activated in the plasma membrane. It may also increase when Ca^2+^ is released from the endoplasmic reticulum by IP3R or RyR. Subsequently, CaM forms a complex with Ca^2+^ and triggers the activation of CaMKK, which in turn exerts its influence on CaMK I, and CaMK IV. CREB, cAMP response element-binding protein; IP3, inositol trisphosphate; IP3R, IP3 receptor; RyR, ryanodine receptors.

Various physiological functions, such as synaptic plasticity, neuronal morphogenesis, and transcriptional activation via the phosphorylation of transcription factors, including cAMP-response element binding protein, have been demonstrated to be regulated by CaMKK-mediated CaMK I and CaMK IV activation. Thus, “CaMK cascade” is the name given to the signal transduction mechanism that is facilitated by the Ca^2+^-dependent kinase cascade (Figure 1).

Over the years, our lab has been engaged in relevant research into the mechanism through which Suanzaoren decoction (SZRD) acts as an antidepressant [14,15,16]. Our previous evidence-based studies have shown that SZRD can reduce the release of inflammatory factors and can improve disorders related to neuronal synaptic remodeling in a rat model of depression caused by chronic stress [17,18], and we have shown that it exerts its antidepressant effects by stimulating the production of neurotrophic factors in the hippocampus [19] and perhaps other brain regions. However, the mechanism through which SZRD has an effect on depression is not well understood. In this study, we used a chronic, unexpected, mild stress (CUMS) rat model to study how SZRD affects the hippocampus by altering the expression of critical components in the CaMK signaling pathway. In order to establish a novel clinical therapy and to lay the foundation for the use of SZRD in the treatment of depression, we sought to clarify the antidepressant impact of SZRD and determine whether SZRD exerts its antidepressant mechanism via the CaMK signaling pathway.

## 2 Materials and methods

### 2.1 Animals

This study used 120 6-week-old specific pathogen-free male Sprague–Dawley rats weighing 190 ± 10 g that were purchased from the Laboratory Animal Center of Heilongjiang University of Chinese Medicine (Laboratory Animal License No. SYXK [black] 2015121002). To establish the CUMS rat model, the animals were acclimated for 1 week in a pathogen-free environment with a room temperature of 20–25°C, a relative humidity of 50–55%, a 12-h light–dark cycle, and free access to food and water. The guidelines for the care and use of laboratory animals published by the National Institute of Health were followed in all aspects of animal care and experiments, and we made all efforts to ensure that animal suffering was minimized. During the experiment, 20 rats in the normal control group did not receive any stimulation, and the other rats were kept in separate cages. Animal Experiment Center at Heilongjiang University of Chinese Medicine provided the specialized rat diet needed for the study. All the feed was subjected to high-pressure sterilization.

### 2.2 Induction of depression by CUMS

**Figure 2:**
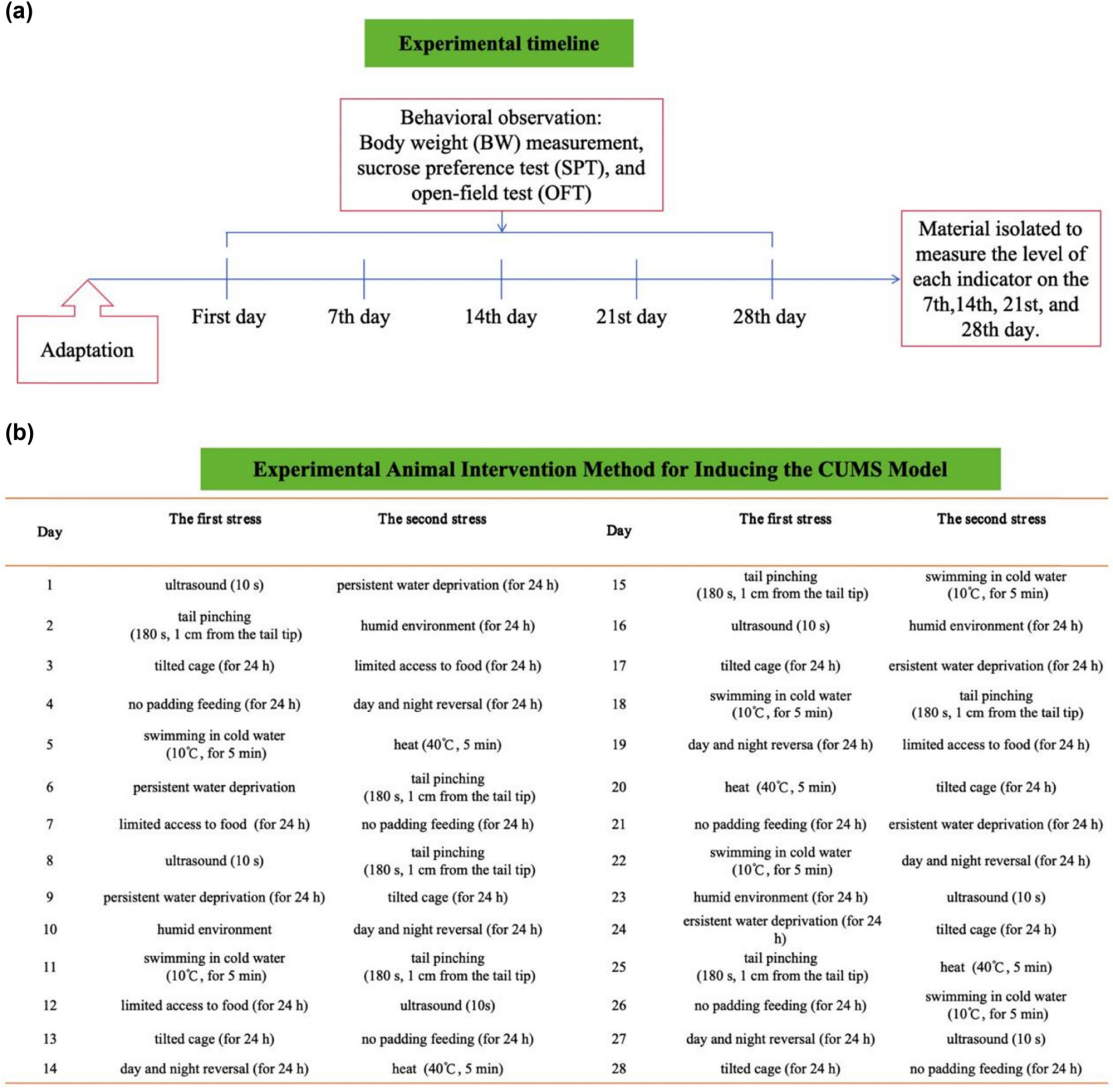
(a) Experimental procedures and (b) the experimental animal intervention method. Before the start of the trial, all rats were fed ad libitum for a full week. All rats were raised in isolation with the exception of the control group, and all rats in the CUMS, SZRD + CUMS, and fluoxetine + CUMS groups were subjected to the CUMS protocol to induce depression. Over the course of the experiment, the CUMS rats were exposed to a variety of stimuli from 8:00 to 9:00 a.m. every day for 28 days. The SZRD doses were 2.5, 5, and 10 g/kg, which were equivalent to 1/2, 1, and 2 times the equivalent clinical dose, respectively. Rats in the fluoxetine + CUMS group were given the drug (1.8 mg/kg, dissolved in a concentration of 90% normal saline) once daily 1 h before being exposed to the stress stimulus. BW, body weight; SPT, sucrose preference test; OFT, open-field test; and CUMS, chronic unpredicted mild stress.

The depression model was created using the same techniques described by Willner et al. [20] and Hennessy et al. [21]. All rats were kept in solitary isolation during the establishment of the rat depression model. Over the 28 days of the experiment, ten stress factors were applied by the method of random arrangement except for the normal control group. Diverse stimuli were introduced to the rats, including high-pitched noise for 10 s, swimming in cold water (10℃ for 5 min), exposure to heat (40℃ for 5 min), tail pinching (180 s, 1 cm from the tail tip), exposure to a humid environment (for 24 h), restricted food (for 24 h), limited potable water (for 24 h), no padding feeding(for 24 h), day and night reversal (for 24 h), and a tilted cage (for 24 h). Two kinds of stimulations were randomly given to the rats every day so that the rats could not foresee the occurrence of stimuli and thus avoid adaptation. Each stress stimulation experiment was performed in a separate laboratory away from the rat’s cage (Figure 2).

### 2.3 Drugs

SZRD is composed of five kinds of Chinese herbal medicines, including Suanzaoren_(stir-fried, 20 g)_, Fuling_(10 g)_ (Poria, Hoelen), Chuanxiong_(10 g)_ (Rhizoma Chuanxiong, Ligusticum), Zhimu_(10 g)_ (Rhizoma Anemarrhenae, Anemarrhena), and Gancao_(5 g)_ (Radix Glycyrrhizae, Licorice), all of which are recorded in the Chinese Pharmacopoeia (Version 2010). The dosage mainly comes from “Jin GuiYaoLue” (Synopsis of Prescriptions of the Golden Chamber) written by Zhang Zhongjing (AD 152–219) at the end of the Han Dynasty. The decoction was purchased by prescription from the pharmacy of the First Affiliated Hospital of Heilongjiang University of Chinese Medicine. All the drugs were mixed in the appropriate proportions, mashed, and soaked in water for 40 min according to the conventional method and then boiled twice, mixed, and filtered [22]. The human dose of SZRD is 55 g/D, and the equivalent dose of 55 g/D in rats is obtained according to the body surface area exchange algorithm between rats and humans 55 × 0. 018 × 5 ≈ 5 g/(kg day). This dose is the clinically equivalent dose; that is, the low, medium, and high doses of SZRD were 2.5, 5, and 10 g/kg, respectively, which were equivalent to 0.5, 1, and 2 times the clinically equivalent dose, respectively.

Fluoxetine was purchased from Lilly Suzhou Pharmaceutical Company Limited (National medicine permission number: J20080016; batch number: 4501A) and came in an aluminum package with 7 tablets/box and 20 mg per tablet. The dosage for adults is 20–60 mg, and the clinically equivalent dose of fluoxetine in rats is 0.04 × 0.018 × 5 ≈ 0.0036 g/kg.

### 2.4 Experimental intervention

A total of 120 healthy male SD rats were randomly divided into the following six groups: control group, CUMS group, SZRD + CUMS low-dose group, SZRD + CUMS medium-dose group, SZRD + CUMS high-dose group, and fluoxetine + CUMS group. All the drugs were administered by gavage.

Control group: The rats in the control group received no outside stimulation and had unrestricted access to food and water.

CUMS group: The aforementioned procedure was used to induce depressive symptoms in the CUMS group rats.

SZRD + CUMS low-dose group: Starting from the first day of modeling, 30 min before each stress (8:00–9:00 a.m. every day), a low dose of SZRD (0.5 g/ml) was administered, which was equivalent to half the dose for a 60 kg adult. This was given once a day for 28 days.

SZRD + CUMS medium-dose group: Starting from the first day of modeling, 30 min before each stress (8:00–9:00 a.m. every day), a medium dose of SZRD (1 g/ml) was administered, which was equivalent to the dose for a 60 kg adult. This was given once a day for 28 days.

SZRD + CUMS high-dose group: Starting from the first day of modeling, 30 min before each stress (8:00–9:00 a.m. every day), a high dose of SZRD (2 g/ml) was administered, which was equivalent to twice the dose for a 60 kg adult. This was given once a day for 28 days.

Fluoxetine + CUMS group: Starting from the first day of modeling, 30 min before each stress (8:00–9:00 a.m. every day), 10 ml/kg dose of 0.36 mg/ml fluoxetine suspension was administered, which was equivalent to the dose in 60 kg adults. This was given once a day for 28 days.

### 2.5 Reagents and antibodies

The following reagents and antibodies were used: A CaM antibody (Affinity, batch number: 6353, 50 μl, 1:500), a CaMK I antibody (Abcam, batch number: AB68234, 100 μl, 1:5,000), a CaMK IV antibody (CST, batch number: 4032S, 100 μl, 1:1,000), and Fura-2/AM (Abcam, batch number: AB120873).

### 2.6 Behavioral observation

All behavioral studies were conducted in complete silence. The same observer, who was unaware of the group assignments, conducted all of the behavioral studies. At the conclusion of the trial, BW was measured, and the SPT and the OFT were administered within 12 h of the last stress exposure.

#### 2.6.1 BW

To evaluate dietary preferences and health, we measured changes in BW from the beginning of the study to day 28.

#### 2.6.2 SPT

Anhedonia were determined using the SPT. For 24 h, the rats were denied both food and water. They were then given 24 h of unrestricted access to two pre-weighed bottles, one containing 150 ml of sucrose solution (1% w/v) and the other containing 150 ml of purified water. To minimize the impact of side preference, we rotated the two solution bottles after 12 h. After 60 min, the two bottles were weighed, and the sugar water preference was calculated as ([sugar water consumption/total liquid consumption] × 100%). The SPT was conducted on day 0 before the intervention and then 28 days after the intervention. Depression was indicated by a decrease in sugar intake.

#### 2.6.3 OFT

Each rat’s locomotor performance was evaluated in a well-lit open-field setup. The open-field equipment consisted of a square arena of 100 cm × 100 cm × 80 cm, with white lines delineating 25 identical squares. To begin, we put each rat gently in the middle of the open-field device and let it freely wander about and investigate its surroundings for 5 min. To measure the rat’s mobility and curiosity, we counted the number of squares it entered (defined as the number of times a horizontal line was crossed by all three paws being in the same square) and the number of times it stood up straight. Each rat’s score was tallied based on how many times it crossed the apparatus (one point was awarded for every time three or four paws entered the same square) and how many times it stood (one point was awarded for every time the rat’s front paws departed the ground simultaneously). After every test, the open-field apparatus was disinfected with 75% ethyl alcohol to remove any residual scent signal. On days 0, 7, 14, and 21, the rats were put through the OFT. The horizontal score reflects the animal’s activity, and the vertical score reflects the animal’s exploration of new and exotic environments.

#### 2.6.4 Tissue collection

On day 28, after the behavioral tests were completed, the rats in each experimental group received an intraperitoneal injection of 10% pentobarbital (0.3 ml/100 g, intraperitoneally) to induce anesthesia. Immediately following sedation, the rats’ heads were severed. Their brains were removed and placed on ice, and the hippocampal tissues from both the left and right cerebral hemispheres were rapidly separated and placed in a freezing tube for rapid cryopreservation in liquid nitrogen for subsequent experimental testing.

#### 2.6.5 Determination of hippocampal Ca^2+^ concentration by the Fura-2/AM method

The hippocampal tissue suspensions were prepared according to the method of Dildy F [22]. The fresh hippocampus were immediately stripped of meninges and blood vessels and then gently cut into pieces. The hippocampal tissue suspensions were prewarmed at 37°C for 5 min, and Fura-2/AM were added (final concentration 2.5 μmol/l). The excitation wavelength and emission wavelength of Fura-2 in the standard solution, loading solution, and hippocampus were measured by scanning (under normal conditions, the excitation wavelength = 340 nm, and 380 nm, and the emission wavelength = 510 nm). Formula of calculation:

\[{{[}{\text{Ca}}^{2+}]}_{i}=\text{Kd}\times (R-{R}_{\min })\times {({R}_{\max }-R)}^{-1}.]\]

#### 2.6.6 Determination of CaM activity

To the suspension of hippocampal tissues was added 1 ml of 70% ethanol and kept overnight at 4°C. The mixture was wash three times with calcium-free PBS, and then 2 ml of tetrafluoropropanol was added, shaked, and exposed to 250–256 nm ultraviolet light for 20 min. Then, they were rinsed with calcium-free PBS, centrifuged at 1,500 rpm for 5 min, and suspended in calcium-free PBS. CaM activity can be detected by flow cytometry detection.

#### 2.6.7 Western blot analysis

The rats were decapitated and their brain tissues were removed 7 days after immunohistochemistry (IHC). The every piece of brain tissue homogenate was centrifuged at 16,000 × *g* for 10 min to remove the supernatant, and the BCA was used to quantify the protein levels. The proteins were separated by SDS-PAGE and transferred onto a PVDF membrane. The membranes were treated with a 5% solution of skim dry milk at room temperature for 2 h to prevent non-specific binding. CaM antibody, CaMK I antibody, and CaMK IV antibody were treated with the membrane overnight at 4°C. Image J (NIH, Bethesda) was used to measure the optical density of the target band after ECL detection of the fluorescence signals.

#### 2.6.8 Real-time quantitative PCR

Trizol (Invitrogen) was used to lyse the cells, and the EZ-10 total RNA extraction kit (Sangon Biotech, Shanghai, China) was used to recover the RNA. The A260/A280 absorbance ratio was used to quantify RNA purity. In addition, an agarose gel was run to ensure the RNA integrity. Reverse transcription and cDNA synthesis were carried out following the protocols outlined in the Revert-Aid Kit and the cDNA Synthesis Kit (Promega GoTaq). The ABI 7500 PCR system (Applied Biosystems) was used to run the PCR reactions in accordance with the manufacturer’s directions for the fluorescent quantitative real-time PCR kit (Qiagen). β-Actin was used as the internal standard (Table 1). The primers are shown in Table 1.

**Table 1:** Sequences of primers (5′−3′) for qPCR: Primer sequences used for quantitative PCR

Target gene	Polarity	Primer sequence (5′ → 3′)	Size (bp)
**Target protein**			
CaM	Forward	GAC TGT CAT GCG GTC ACT GG	20
	Reverse	TGA CCT GTC CGT CTC CAT CAA T	22
CaMK I	Forward	GAA TTC TAT GCC AGG GGC AGT GGA AGG C	28
	Reverse	GGA TCC AAC TGC TCA CTC ACT GAC TGG	27
CaMK IV	Forward	GCG GCT AGC ATG CTC AAA GTC ACG	24
	Reverse	CCT CCT CTT GCA	12
**Housekeeping gene**			
β-Actin	Forward	AGC GGG AAA TCG TGC GTG	18
	Reverse	CGT GGT GGT ACA TGG GAC	18

#### 2.6.9 IHC

The frozen cerebral tissues were fixed, embedded, sliced, baked, and dewaxed [23]. Endogenous peroxidase activity was inhibited by incubating the sections in a fresh 3% H_2_O_2_ solution at 25°C for 10 min, after which the sections were rinsed three times for 3 min in 0.01 M PBS. The sections were treated with goat serum working solution for 20 min at room temperature and then incubated with the primary antibody overnight at 4°C. The sections were washed in 0.01 M PBS three times for a total of 3 min each time. After a 30-min incubation in biotinylated secondary antibodies at 37°C, the sections were rinsed three times in 0.01 M PBS for 3 min each and developed with DAB developer. After sealing the slices with a neutral gum, they were counterstained with hematoxylin and studied under an optical microscope.

## 3 Statistical analysis

SPSS 26.0 was used for all statistical analyses (IBM, Armonk, New York, NY, USA). The mean values and standard deviations of all data sets are presented. One-way analysis of variance was used for comparison among multiple groups, and Tukey’s post hoc tests were used to verify. A value of *p* < 0.05 was considered statistically significant. The histograms were generated using GraphPad Prism 9.0 software (GraphPad Software, Inc.).


**Ethical approval:** The research related to animals’ use has been complied with all the relevant national regulations and institutional policies for the care and use of animals. The animal study was reviewed and approved by the Animal Ethics Committee of Heilongjiang University of Chinese Medicine (Laboratory Animal License No. SYXK (black) 2015121002).

## 4 Results

### 4.1 Effect of SZRD treatment on behavior in CUMS model rats

#### 4.1.1 Changes in BW

**Figure 3:**
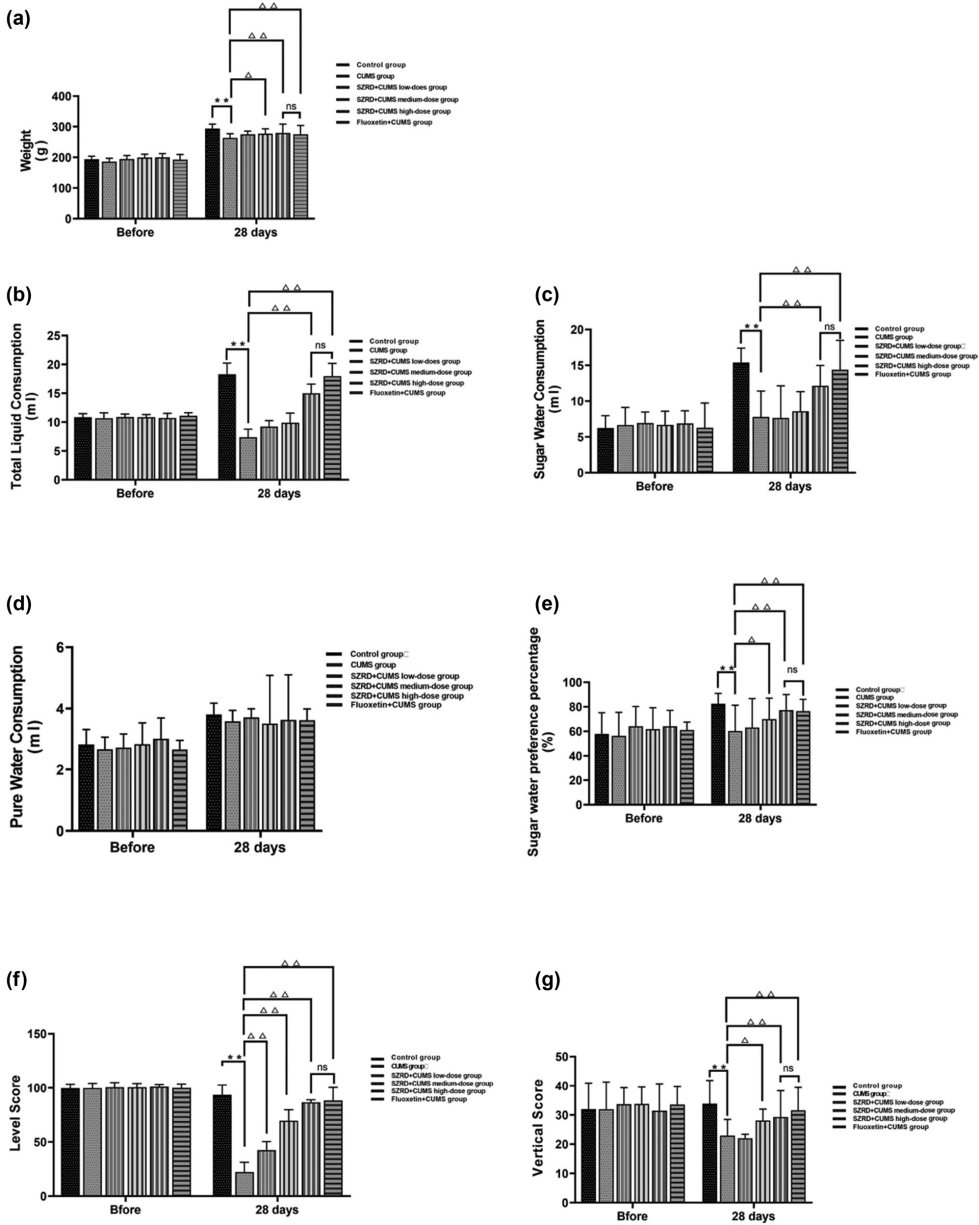
The effect of SZRD treatment on behavior in CUMS model rats. (a) BW. (b) Total liquid consumption (ml). (c) Sugar water consumption (ml). (d) Pure water consumption (ml). (e) Sugar water preference (%). (f) Horizontal score. (g) Vertical score. ^*^ *p* < 0.05, ^**^ *p* < 0.01 vs control; ^△^ *p* < 0.05, ^△△^ *p* < 0.01 vs CUMS. *n* = 20 per group.

Before the intervention, there were no significant differences in BW between any of the groups. After 28 days of modeling, the CUMS group showed significant weight loss compared with the control group (^**^ *p* < 0.01 vs control group). After SZRD and fluoxetine treatment, compared with the CUMS group, the BW of dates of the SZRD + CUMS medium-dose high-dose groups and the fluoxetine + CUMS group increased (^Δ^ *p* < 0.05, ^ΔΔ^ *p* < 0.01 vs CUMS group). However, there was no significant difference (*p* > 0.05) in BW between the SZRD + CUMS high-dose group and the fluoxetine + CUMS group, indicating that SZRD had the same effect as fluoxetine in the treatment of CUMS model rats when it reached a certain concentration (Figure 3a).

#### 4.1.2 Changes in SPT

Before the intervention, there were no significant differences in SPT between the groups. After 28 days of modeling, the intake of total liquid consumption, sugar water consumption, and sugar water preference percentage in the CUMS group decreased significantly (^**^ *p* < 0.01 vs control group). In the SZRD + CUMS medium and high-dose groups, the total liquid consumption, sugar water consumption, and sugar water preference percentage increased between SZRD + CUMS high-dose group and fluoxetine + CUMS group (^Δ^ *p* < 0.05, ^ΔΔ^ *p* < 0.01 vs CUMS group). There was no significant change in the SZRD + CUMS low-dose group. When SZRD reached a certain concentration, the therapeutic effect in the treatment of CUMS model rats was similar to that of fluoxetine (*p* > 0.05) (Figure 3b–e).

#### 4.1.3 Changes in OFT

Before the intervention, there were no significant differences in OFT between the groups. After 28 days of modeling, the horizontal score and vertical score of the CUMS group decreased (^**^ *p* < 0.01 vs control group). After treatment with SZRD and fluoxetine, the horizontal score and vertical score of each SZRD + CUMS dose and the fluoxetine + CUMS group were increased (^Δ^ *p* < 0.05, ^ΔΔ^ *p* < 0.01 vs CUMS group). For the vertical score, only the SZRD + CUMS high-dose group and fluoxetine + CUMS group increased (^Δ^ *p* < 0.05, ^ΔΔ^ *p* < 0.01 vs CUMS group). We concluded that when SZRD reached a certain concentration, the OFT score of the CUMS model rats was significantly changed, and the therapeutic effect was similar to fluoxetine (*p* > 0.05) (Figure 3f–g).

### 4.2 Results of Ca^2+^ concentration determination in the hippocampus of each group

**Figure 4:**
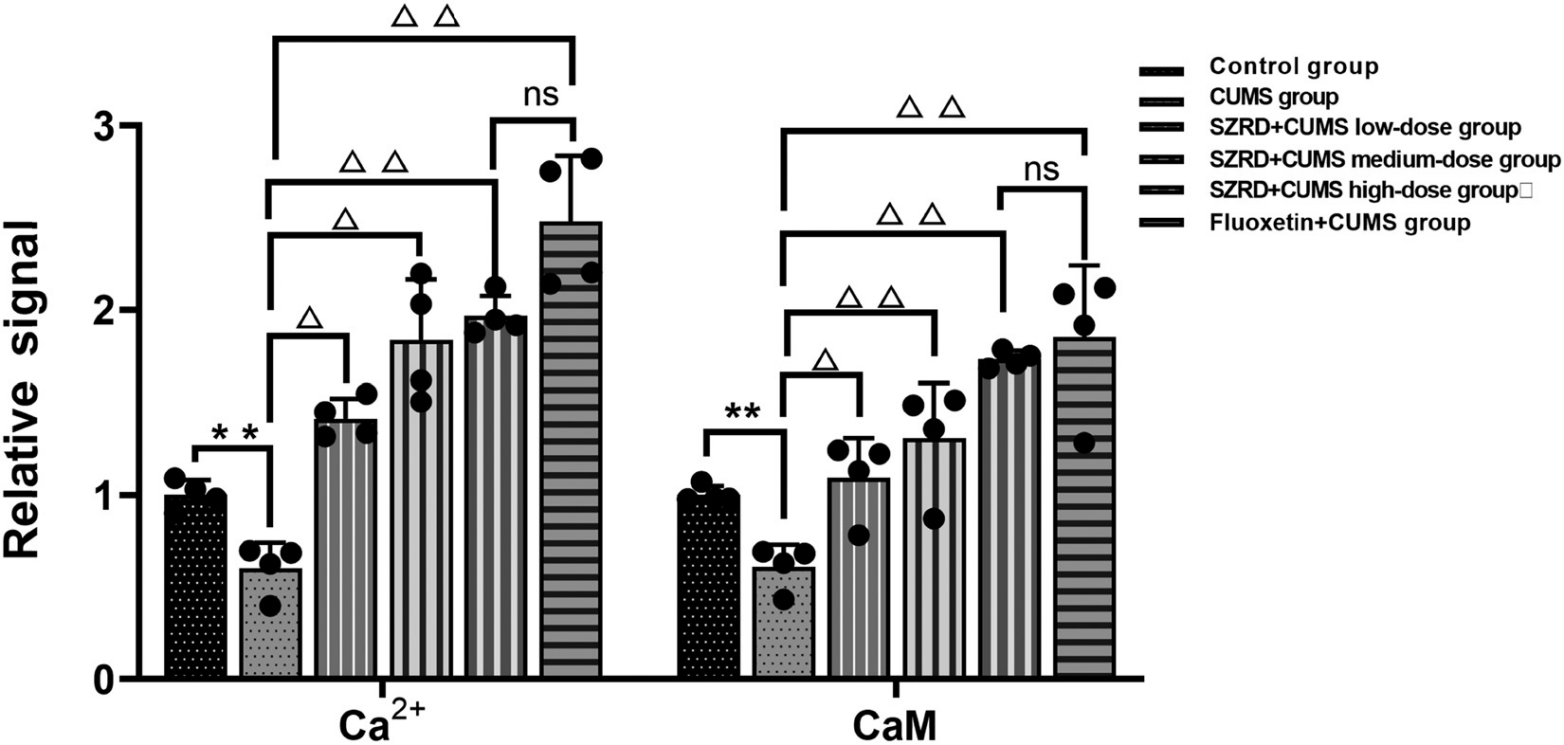
Ca^2+^ titer assay and CaM activity assay. The data were normalized to the control group. ^*^ *p* < 0.05, ^**^ *p* < 0.01 vs control; ^△^ *p* < 0.05, ^△△^ *p* < 0.01 vs CUMS; *n* = 4 per group.

The Fura-2/AM method was used to determine the relative concentration of Ca^2+^ in the hippocampus (Figure 4). From the experimental results, after 28 days, compared with the control group, the Ca^2+^ titer of CUMS group was relatively low (^**^ *p* < 0.01 vs control group). After SARD and fluoxetine intervention, the Ca^2+^ relative titer of SZRD + CUMS each dose groups and fluoxetine + CUMS group was relatively increased (^Δ^ *p* < 0.05, ^ΔΔ^ *p* < 0.01 vs CUMS group). There was no significant difference between the SZRD + CUMS high-dose groups and the fluoxetine + CUMS group (*p* > 0.05). Similar to fluoxetine treatment, SZRD can promote the influx of Ca^2+^.

### 4.3 Results of CaM activity determination in the hippocampus of each group

Flow cytometry was used to determine CaM activity in the hippocampus. After 28 days, compared with the control group, the CaM activity of CUMS group was relatively low (^**^ *p* < 0.01 vs control group). After SZRD and fluoxetine intervention, the CaM activity of SZRD + CUMS each dose group and fluoxetine + CUMS group was relatively increased (^Δ^ *p* < 0.05, ^ΔΔ^ *p* < 0.01 vs CUMS group). There was no significant difference between the SZRD + CUMS each dose groups and the Fluoxetine + CUMS group (*p* > 0.05). Similar to fluoxetine treatment, SZRD could stimulate Ca^2+^ signal transmission and increase CaM activity (Figure 4).

### 4.4 Effect of SZRD on protein and mRNA expression of CaMK signaling pathway in the hippocampus of CUMS model rats

**Figure 5:**
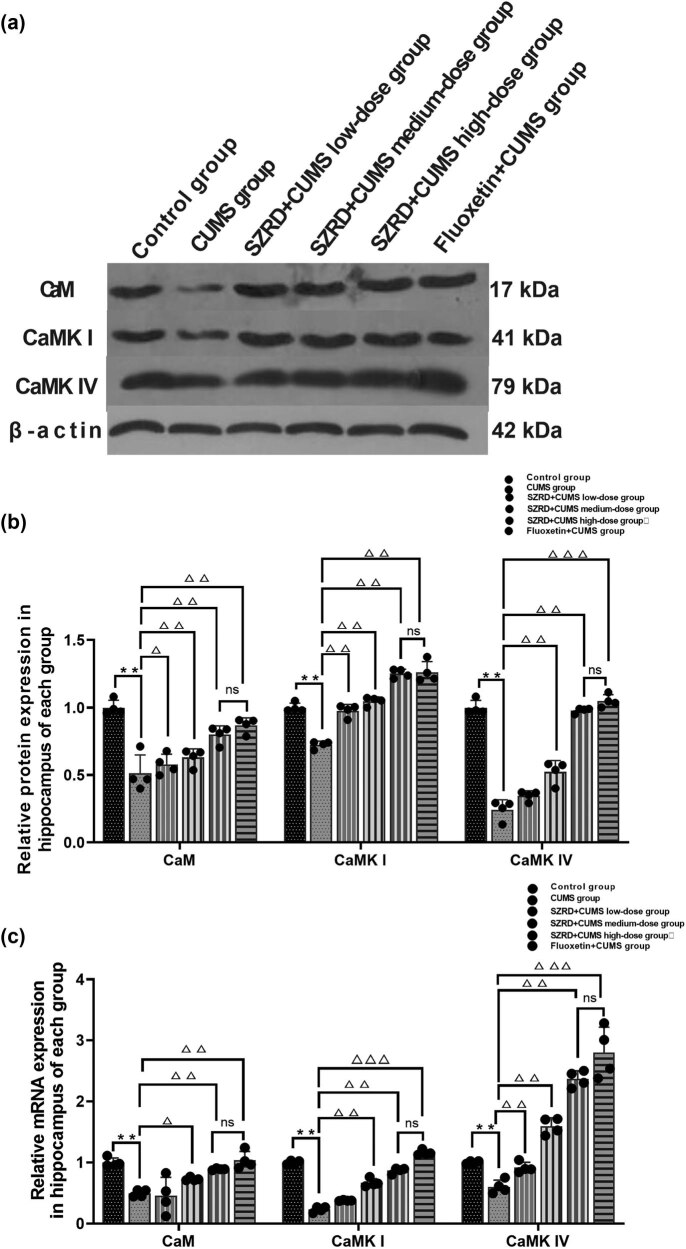
The effects of SZRD treatment on CaMK protein and mRNA expression in the hippocampus at 28 days after CUMS. (a) Western blot analysis of representative bands and (b) quantification of CaM, CaMK I, and CaMK IV expression in the hippocampus. (c) Quantitative analysis of relative CaM, CaMK I, and CaMK IV mRNA expression in the ipsilateral basal ganglia by qPCR. The data were normalized to the control group. The results were consistent with the Western blot trend. ^*^ *p* < 0.05, ^**^ *p* < 0.01 vs control; ^△^ *p* < 0.05, ^△△^ *p* < 0.01 vs CUMS; *n* = 4 per group.

Western blot analysis was used to determine the relative abundance of CaM, CaMK I, and CaMK IV. Western blot results showed that after 28 days of modeling, the protein expression of CaM, CaMK I, and CaMK IV in the hippocampus of the CUMS group was decreased (^**^ *p* < 0.01 vs control group). After SZRD and fluoxetine treatment, the protein expression of CaM, CaMK I, and CaMK IV in the hippocampus of the SZRD + CUMS and fluoxetine + CUMS groups was increased (^Δ^ *p* < 0.05, ^ΔΔ^ *p* < 0.01 vs CUMS group) (Figure 5a and b).

The mRNA expression levels of CaM, CaMK I, and CaMK IV were analyzed by qPCR. The results showed that after 28 days of modeling the mRNA expression of CaM, CaMK I, and CaMK IV in the hippocampus of rats in CUMS group was significantly decreased (^**^ *p* < 0.01 vs control group). After SZRD and fluoxetine treatment, the mRNA expression of CaM, CaMK I, and CaMK IV in the hippocampus of each SZRD + CUMS dose group and the fluoxetine + CUMS group was significantly increased (^Δ^ *p* < 0.05, ^ΔΔ^ *p* < 0.01 vs CUMS group) (Figure 5c).

Moreover, there was no statistical difference between the SZRD + CUMS high-dose groups and the fluoxetine + CUMS group (*p* > 0.05). Similar to fluoxetine treatment, SZRD could alleviate depressive symptoms in CUMS model rats by activating the CaMK signaling pathway.

### 4.5 Effects of SZRD treatment on the expression of CaMK signaling pathway in the hippocampus of CUMS model rats

**Figure 6:**
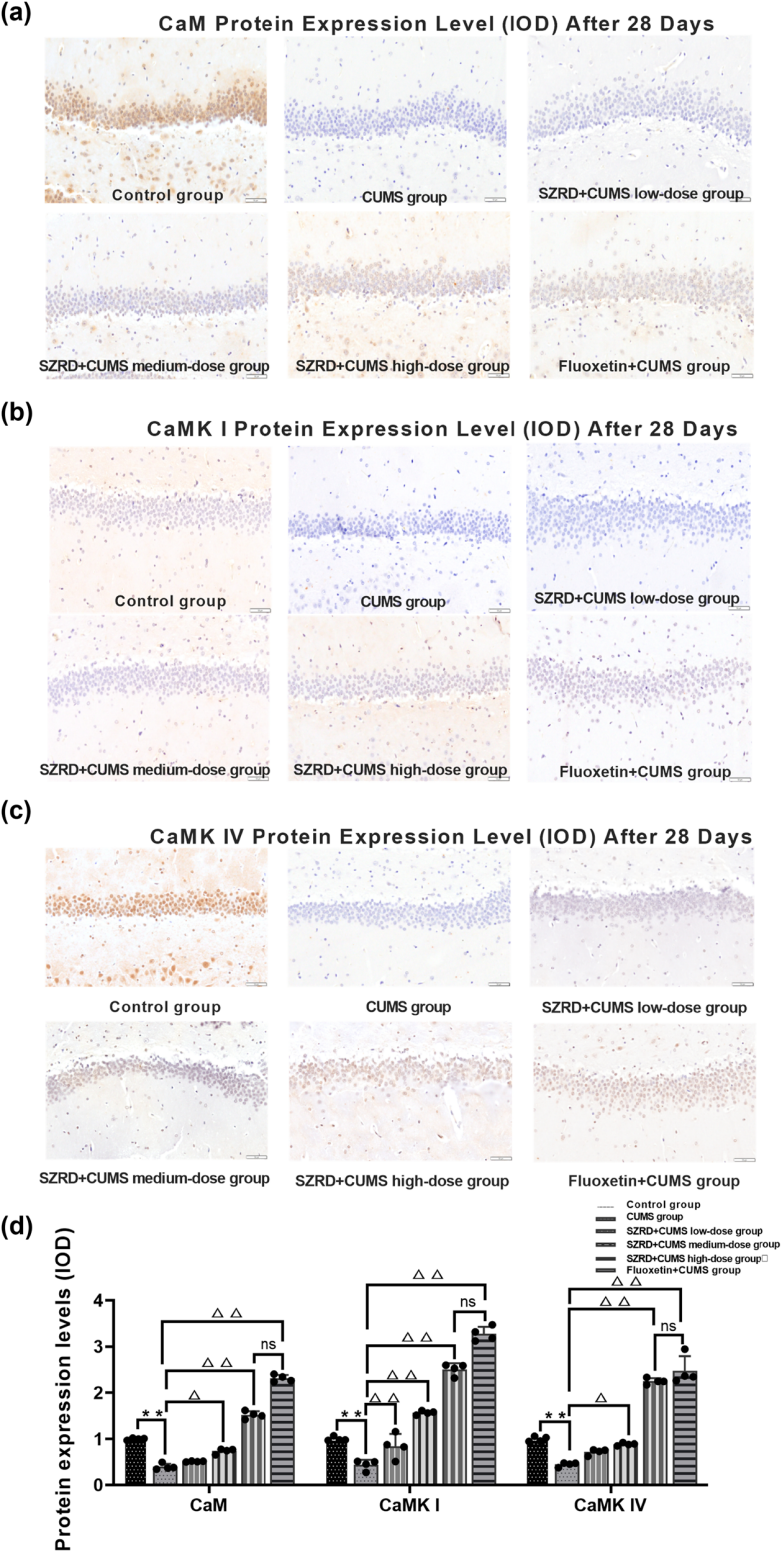
The effect of SZRD treatment on the relative expression levels of various proteins in hippocampus at 28 days after CUMS. (a) CaM protein expression level (IOD) after 28 days. (b) CaMK I protein expression level (IOD) after 28 days. (c) CaMK IV protein expression level (IOD) after 28 days. (d) Histogram of CaM, CaMK I, and CaMK IV IOD values. The data were normalized to the control group. The results were consistent with the Western blot trend. ^*^ *p* < 0.05, ^**^ *p* < 0.01 vs control; ^△^ *p* < 0.05, ^△△^ *p* < 0.01 vs CUMS; *n* = 4 per group.

After 28 days of modeling, the subcellular localization of CaM, CaMK I, and CaMK IV protein expression in the hippocampus was detected by IHC. The results showed that in the control group, CaM was mainly located in the cell membrane; CaMK I was mainly located in the cytoplasm; and CaMK IV was mainly distributed in the nucleus. Moreover, CaM, CaMK I, and CaMK IV less number of positive cells in the CUMS group. After SZRD and fluoxetine treatment, CaM, CaMK I, and CaMK IV the positive expression of the SZRD + CUMS each dose group and fluoxetine + CUMS group increased, and CaM, CaMK I, and CaMK IV subcellular localization was more distributed in the cell membrane, cytoplasm, and nucleus (Figure 6a–c).

Furthermore, immunohistochemical results showed that the integrated optical density (IOD) values of CaM, CaMK I, and CaMK IV in the hippocampus of the CUMS group were decreased (^**^ *p* < 0.01 vs control group). After SZRD and fluoxetine treatment, the IOD values of CaM, CaMK I, and CaMK IV in the hippocampus of rats in each dose of SZRD + CUMS and the fluoxetine + CUMS group were increased (^Δ^ *p* < 0.05, ^ΔΔ^ *p* < 0.01 vs CUMS group). The immunohistochemical results were consistent with the trends seen in the Western blots and qPCR, indicating that the therapeutic effects of SZRD and fluoxetine on the CUMS rats were not statistically significant (*p* > 0.05).

Immunohistochemical results showed that, compared with the control group, the IOD values of CaM, CaMK I, and CaMK IV of the CUMS group were decreased in the hippocampus (^**^ *p* < 0.01 vs control group). After SZRD and fluoxetine treatment, compared with the CUMS group, the IOD values of CaM, CaMK I, and CaMK IV were increased in the SZRD + CUMS each dose group and fluoxetine + CUMS group (^Δ^ *p* < 0.05, ^ΔΔ^ *p* < 0.01 vs CUMS group). Moreover, there was no statistical difference between the SZRD + CUMS high-dose group and fluoxetine + CUMS group (*p* > 0.05). The IOD values were consistent with the subcellular localization results. Similar to fluoxetine treatment, SZRD could improve the subcellular localization of CaM, CaMK I, and CaMK IV and activate the CaMK signaling pathway in order to improve CUMS model of rats' hippocampal cells form (Figure 6d).

## 5 Discussion

The onset of depression is usually gradual and its course is intermittent, but the condition will progress in both the frequency and severity of episodes over time [24]. Depression is not a purely psychological disease, but unlike diabetes, cancer, COPD, etc., where a doctor may simply run a blood test to determine if a patient has the disease, depression cannot be diagnosed by tests of blood chemistry, diagnostic imaging, or surgical biopsies [25]. It is instead diagnosed based on the presence of a diverse array of symptoms, including low mood, mood swings, feelings of inferiority, hopelessness, worthlessness, difficulty focusing and thinking clearly, changes in appetite (resulting in loss of weight or gain), sleep disturbances (whether excessive sleep or excessive wakefulness), low energy, fatigue, increased agitation, decreased interest in pleasurable stimuli (such as sex, food, or social interactions), and recurrent thoughts of suicide. We used a rat model of CUMS-induced depression, measured their BW, and performed the SPT and OFT in order to examine behaviors associated with reward and pleasure sensitivity and anhedonia. Our findings show that CUMS can result in a significant reduction in body mass and reduced intake of sugar solution. Body mass largely determines the amount of sugar solution consumed.

Moreover, the rats of the CUMS group also had trouble adjusting to novel environments, showed little interest in rewarding stimuli, remained relatively stable emotionally, and were more vulnerable to negative states of mind during the entire CUMS process. The findings of the current study are comparable with those of earlier research, which together suggest that CUMS causes depressive-like behaviors and successfully imitates the depressive symptoms seen in human patients [26,27]. In our study, it was shown that SZRD could effectively improve depression-like behavior in CUMS model rats. Because depressive symptoms could be reliably identified by our approach, we may conclude that SZRD has potential clinical use in the treatment of depression.

SZRD was first described in the *Synopsis of the Golden Chamber – Article VI-Pulse, Syndrome and Treatment of Blood Impediment Consumption Disease*, which provides the classic representative formula for treating “consumptive exhaustion and sleeplessness.” Traditional data show that SZRD has good biocompatibility in that it regulates both Yin and Yang and harmonizes both the liver and spleen [28], and it combines convergence with divergence and applies both replenishing and reducing. “Qi” moves smoothly up and down so that it is replenished without stagnation, and the functions of the viscera can be restored. The combination of the various herbs in SZRD nourishes the blood, calms the nerves, clears heat, and eliminates vexation so that fire can be removed and the mind can be relaxed. Multiple studies have demonstrated that SZRD has a crucial role in the treatment of primary insomnia, insomnia following stroke, depression, anxiety, mental disorders associated with COVID-19, and metabolic diseases [29]. Another study showed that SZRD can improve depressive-like behaviors by regulating the microbiota–gut–brain axis by inhibiting the TLR4/NFκB/NLRP3 inflammation signaling pathway [30]. We found in the present study that SZRD significantly reduced depressive symptoms and induced positive behavioral changes. We also found no statistically significant differences between the SZRD high-dose group and the fluoxetine groups, suggesting that SZRD high-dose is similarly effective as fluoxetine. Moreover, it is understood that compared to fluoxetine, the advantage of SZRD is that it does not have serious side effects, while fluoxetine has more toxic side effects, such as activation syndrome, which has been linked to reduced sleep quality [31]. There is also evidence to suggest that initial exposure to fluoxetine increases the risk of some heart abnormalities. CaMKs are multifunctional kinases dependent on Ca^2+^/CaM, and the cascading members are CaMK I, CaMK II, CaMK III, and CaMK IV [32]. The substrates of these proteins include ATP-dependent calcium pumps, various target enzymes, and various calcium channels that can crosstalk with other signaling pathways in cells, thus forming a complex three-dimensional cross-signal transmission network that regulates various adaptive responses after cell stress [33], plays a role in memory, and influences synaptic plasticity in the hippocampus. Their expression levels, subtype compositions, and functions are different in different brain regions, and they regulate a variety of protein substrates. The study of Ca^2+^/calmodulin (CaM)-related mechanisms is crucial for the clinical application of antidepressant drugs. Ca^2+^/CaM are regulators of the cell cycle, and Ca^2+^/CaM binds to CaMKs to activate the enzymes.

CaM, as an intracellular Ca^2+^ receptor, can reversibly bind to Ca^2+^ and further activate downstream Ca^2+^/CaM-dependent protein kinases. Activation of neurons leads to a rise in intracellular Ca^2+^, which in turn sets off a series of Ca^2+^-dependent signaling pathways. Multifunctional CaMKs are essential for neural transmission, synaptic plasticity, circuit formation, and cognitive function, and recent research has shown that CaMKs play a pathophysiological role in neuropsychiatric diseases such as addiction, depression, schizophrenia, and a variety of neurodevelopmental disorders [34]. Members of the CaMK family interact with Ca^2+^ and CaM complexes through different activation mechanisms, and Ca^2+^/CaM binding releases autoinhibitory–autophosphorylation–mediated activation of CaMK I, which is induced by poly CaMK II and CaMKK-dependent phosphoric acid switching. In order to activate the signaling activity of their substrate proteins, CaMKs require the binding of Ca^2+^/CaM to phosphoserine/threonine residues on those proteins.

The major purpose of our study was to investigate the role of SZRD in the treatment of depression by focusing on the low substrate specificity and affinity of “multifunctional” CaMKs such as CaMK I and CaMK IV. Having such a wide range of substrates makes multifunctional CaMKs ideal for their role as potent and multivalent neuronal Ca^2+^ effectors that mediate the changes in brain states that are involved in cognitive and other processes. Both CaMK I and CaMK IV are monomers, and the basic structure between the two is common, similar to CaMK II, except that neither of them has a self-linking domain at their C-terminus. Current research suggests that suppressing CaMK I and CaMK IV expression might cause pathological phosphorylation states in numerous target substrates, which can maintain maladaptive alterations in key neuronal characteristics over the long term. Several investigations have demonstrated the previously unknown function of the CaMK I pathway in the Ca^2+^-dependent regulation of neuronal morphogenesis [35]. In addition, it has been demonstrated that the movement of growth cones is a common feature of axon growth [36], the formation and growth of axon polarity, and the activity of dendrites, and are dependent on nuclear importin-α mediated accumulation of CaMK IV in the nucleus [37]. CaMK IV is a signaling molecule necessary for synaptic plasticity and is involved in different forms of memory, such as spatial memory, fear memory, and some specific motor memory storage and other advanced brain functions [38], especially during long-term potentiation and long-term depression activation [11]. CaMK IV has been shown to be downregulated in the hippocampus of stressed rats, resulting in reduced sucrose preference in the animals, which reflects the state of depression [39]. We confirmed here that the expression of CaMK IV is associated with the pathophysiology of depression and that activation of CaMK IV can regulate the state of depression. In the study by Bai et al., the activity of the CaMK signaling pathway in cases of depression could be regulated through electroacupuncture intervention, and it has been demonstrated that the CaMK signaling pathway plays an important role in depression [34]. Research into the phosphorylation sites and activation routes of CaMKs, as well as the development of a safe, sensitive, and effective method for assessing the ensuing synaptic alterations, seems like a viable direction for future investigations.

This study measured Ca^2+^ concentration, CaM activity, and key proteins of the CaMK signaling pathway. Molecularly, CaM activity is correlated with Ca^2+^ concentration. On the one hand, SZRD could increase the titer of Ca^2+^ in the hippocampus of CUMS model rats. With the increase of Ca^2+^ concentration, it could mediate the signal transmission between hippocampal neurons, cause the formation of Ca^2+^/CaM complex, activate the “CaM-kinase cascade,” and promote the signal transmission between CaMK I and CaMK IV in hippocampal neurons. On the other hand, SZRD could improve the subcellular localization of key proteins in the CaMK signaling pathway (CaM, CaMK I, and CaMK IV), mediate the intercellular transport of Ca^2+^, and promote the release of neurotransmitters from presynaptic neurons to postsynaptic neurons. Moreover, calcium influx was one of the important factors in the generation of action potential, which could trigger the change of intracellular potential and trigger the generation of action potential. In terms of behavioral symptoms, the main symptoms of CUMS model rats, such as depressive-like behavior and restlessness, were gradually alleviated according to the changes in the SZRD dose–effect relationship.

In conclusion, treatment with SZRD can be performed with little effort. It can reduce the number of times people turn to antidepressants, saving money for both the patients and society. This research suggests that SZRD exerts its antidepressant effect via modulation of central components of the CaMK signaling pathway. The antidepressant effect of SZRD needs to be studied further to ascertain the mechanism underlying its effects and to determine if it is related to epigenetic changes in hippocampal synapse regeneration, which could lead to novel approaches to the treatment of depression.
